# Osteosarcopenia and trabecular bone score in patients with type 2 diabetes mellitus

**DOI:** 10.20945/2359-3997000000418

**Published:** 2021-11-11

**Authors:** Luciana Muniz Pechmann, Ricardo R. Petterle, Carolina A. Moreira, Victoria Z. C. Borba

**Affiliations:** 1 Universidade Federal do Paraná Hospital de Clínicas Centro de Diabetes Curitiba Curitiba PR Brasil Divisão de Endocrinologia (Serviço de Endocrinologia e Metabologia do Paraná – SEMPR), Hospital de Clínicas da Universidade Federal do Paraná e Centro de Diabetes Curitiba, Curitiba, PR, Brasil; 2 Universidade Federal do Paraná Faculdade de Medicina Setor de Ciências da Saúde Curitiba PR Brasil Setor de Ciências da Saúde, Faculdade de Medicina, Universidade Federal do Paraná, Curitiba, PR, Brasil; 3 Universidade Federal do Paraná Hospital de Clínicas Divisão de Endocrinologia Curitiba PR Brasil Divisão de Endocrinologia (Serviço de Endocrinologia e Metabologia do Paraná – SEMPR), Hospital de Clínicas, Universidade Federal do Paraná, Curitiba, PR, Brasil

**Keywords:** Sarcopenia, type 2 diabetes mellitus, muscle weakness, fractures, osteoporosis

## Abstract

**Objective:**

To evaluate the prevalence of osteosarcopenia and the association of osteosarcopenia with trabecular bone score (TBS) in a group of patients with type 2 diabetes mellitus(T2DMG) compared with a paired control group (CG).

**Materials and methods:**

Cross-sectional study with men and women ≥ 50 years recruited by convenience. Patients in both groups answered questionnaires and underwent evaluation of bone mineral density (BMD), handgrip strength (HGS), and TBS. The T2DMG also underwent a gait speed (GS) test. Sarcopenia was defined as low lean mass plus low HGS or GS according to the Foundation for the National Institute of Health Sarcopenia Project, and osteosarcopenia was deemed present when sarcopenia was associated with osteopenia, osteoporosis, or low-energy trauma fractures.

**Results:**

The T2DMG (n = 177) and CG (n = 146) had, respectively, mean ages of 65.1 ± 8.2 years and 68.8 ± 11.0 years and 114 (64.4%) and 80 (54.7%) women. T2DMG versus the CG had higher rates of osteosarcopenia (11.9% versus 2.14%, respectively, p = 0.010), sarcopenia (12.9% versus 5.4%, respectively, p < 0.030), and fractures (29.9% versus 18.5%, respectively, p = 0.019), and lower HGS values (24.4 ± 10.3 kg versus 30.9 ± 9.15 kg, respectively, p < 0.001), but comparable BMD values. Mean TBS values were 1.272 ± 0.11 and 1.320 ± 0.12, respectively (p = 0.001). On multivariate analysis, age, greater waist circumference, fractures, and osteoporosis increased the risk of degraded TBS. Osteosarcopenia was associated with diabetes complications (p = 0.03), calcium and vitamin D supplementation (p = 0.01), and all components of osteosarcopenia diagnosis (p < 0.05).

**Conclusion:**

Compared with the CG, the T2DMG had a higher prevalence of osteosarcopenia, sarcopenia, and fractures and lower bone quality assessed by TBS.

## INTRODUCTION

Type 2 diabetes mellitus (T2DM) affects bone and muscle health. Osteoporosis, fractures, sarcopenia, and T2DM are common in the elderly population, and recent evidence has indicated common pathophysiological pathways linking T2DM to osteoporosis and sarcopenia (1). Bone, muscle, and glucose metabolism are intimately intertwined in a complex network (2). Indeed, patients with T2DM with poor glycemic control and chronic complications have an increased risk of fractures, despite greater bone mineral density (BMD) compared with individuals without T2DM (3,4).

This apparent contradiction of increased BMD associated with a greater risk of fracture in patients with T2DM is probably due to poor bone quality undetected by BMD measurement (4,5). This observation highlights the importance of assessing bone strength and fracture risk with methods other than BMD, such as trabecular bone score (TBS), an indirect measure of bone quality (6). Indeed, low TBS values have been associated with fractures in patients with T2DM (7-9). Additionally, insulin appears to affect bone quality and quantity in patients with T2DM. Hyperinsulinemia associated with insulin resistance and visceral obesity – all frequent in patients with T2DM – correlate with increased bone mass but deteriorated bone quality and muscle strength and loss of muscle function (10).

In terms of muscle quality and function, sarcopenia occurs earlier and 3-16 times more frequently in individuals with diabetes compared with those without diabetes. Additionally, patients with sarcopenia and T2DM are predisposed to long-term diabetes complications, frailty, hospitalizations, and premature death (10,11).

The link between sarcopenia and osteoporosis remains elusive, owing mostly to lack of a standardized definition for sarcopenia and adoption of different criteria defining this condition across studies (12,13). Still, sarcopenia and osteoporosis coexist in many individuals, a condition known as osteosarcopenia. Compared with patients with sarcopenia or osteoporosis alone, those with osteosarcopenia have worse health-related outcomes regarding falls, fractures, physical performance, quality of life, and mortality (13).

Only a few studies to date have assessed the effect of sarcopenia on fractures and bone quality in T2DM, while the impact of comorbidities and chronic complications on the development of osteosarcopenia in this population remains unknown (13-15). Also, the prevalence of this condition in patients with T2DM is still unclear despite the critical impact of osteosarcopenia on health. Based on these considerations, the aims of this study were to investigate the prevalence of osteosarcopenia and the association between osteosarcopenia and bone microarchitecture assessed by TBS in patients with T2DM compared with controls.

## MATERIALS AND METHODS

This was a cross-sectional, controlled study in patients with T2DM treated at the research center of a tertiary hospital. The study protocol was approved by the institution’s ethics committee (number 53569116.7.0000.0096).

### Study sample and data collection

**Figure 1:**
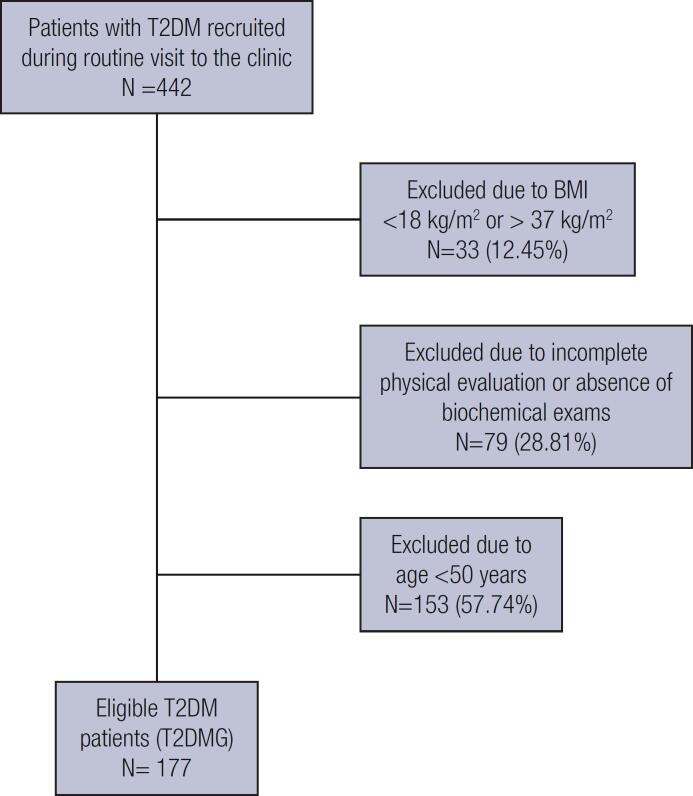
Flowchart of the selection process of patients included in the type 2 diabetes mellitus (T2DM) group.

Men and women (≥50 years) with treated T2DM diagnosed for ≥1 year were recruited by convenience during routine visits to the outpatient clinic of *Serviço de Endocrinologia e Metabologia do Hospital de Clinicas da Universidade Federal of Paraná* (SEMPR). Patients were selected during this visit after inclusion and exclusion criteria were evaluated and comprised the T2DM group [T2DMG]) (Figure 1). Assessments were performed on the day of the medical appointment between March to October 2019.

Community-dwelling healthy volunteers belonging to the SEMPR database with normal gait and without T2DM or glucose intolerance comprised the control group (CG). CG were matched by age and sex with patients in the T2DMG using the frequency matching method.

The study excluded professional athletes; patients unable to understand the study protocol, immobilized, or disabled; individuals with body mass index (BMI) > 37 kg/m² or < 18 kg/m²; patients with chronic diseases under current treatment that could interfere with the assessment of the variables and those receiving hormone replacement therapy, medications affecting muscle mass or body composition (*e.g.*, diuretics, corticosteroids), and protein supplements.

### Questionnaire

A structured questionnaire developed for the study collected baseline disease characteristics, treatment, comorbidities, lifestyle habits, amount of dietary calcium intake from dairy foods, and a self-reported history of low-energy trauma fractures. Calcium or vitamin D supplementation was evaluated as present or absent. Daily calcium intake was classified based on dietary recommendations (recommended amount > 1,200 mg) (16). Insulin use and diabetes complications (retinopathy and cataract, distal motor sensory peripheral neuropathy, nephropathy, and cardiovascular disease) were retrieved from medical records and confirmed by the patients’ physicians.

### Anthropometric measurements

Weight was measured using a scale with accuracy of 100 g and capacity ≤150 kg (*Indústrias* Filizola SA, São Paulo, Brazil). Height was measured with a Tonelli stadiometer with 0.1 cm accuracy (IN Tonelli SA, Santa Catarina, Brazil). BMI classification followed the World Health Organization (WHO) recommendations (17). Waist circumference (WC) was obtained in the largest abdominal perimeter between the last rib and the iliac crest, values ≥ 102 cm and ≥ 88 cm in men and women, respectively, were considered increased (18).

### Evaluation of body composition and BMD

Body composition (BC) and BMD were assessed using the dual-energy x-ray absorptiometry (DXA) system Lunar Prodigy equipped with the software enCORE, v18 (GE Medical Systems, Madison, WI, USA). Total body composition (BC), lumbar spine (L1-L4), total hip, and femoral neck BMD were analyzed following the International Society for Clinical Densitometry guidelines (19). BC parameters were further analyzed by sex. Precision coefficients were 1.30 % for lumbar spine, 1.28 % for femoral neck, and 0.56 % for total femur. BMD categories were classified according to the WHO criteria (20) as normal, osteopenia, and osteoporosis. DXA was performed by a certified operator, and the results were analyzed by a certified radiologist. The sum of arm and leg lean mass obtained from the BC analysis yielded the appendicular lean mass (ALM).

### Previous nonvertebral fractures

Self-reported history of low-energy trauma fractures occurring after 50 years of age excluding skull, toes, and face fractures, were categorized as absent or present.

### TBS

TBS was measured from lumbar spine (in L1-L4 vertebrae not excluded from the BMD measurement) DXA scans using TBS iNsight, v3.0 (MediMaps, Geneva, Switzerland). The values were stratified according to those published in studies including Latin American populations. According to the DXA manufacturer, the microarchitecture was considered degraded with values ≤ 1.230, partially degraded with values > 1.230 and < 1.310, and normal with values ≥ 1.310 (21).

### Muscle mass

Muscle mass (MM) was calculated as the ratio of ALM to BMI values, and low MM (LMM) was defined as MM < 0.789 in men and < 0.512 in women, according to FNIH criteria. Total lean mass (TLM), obtained in the BC assessment, was also analyzed according to sex.

### Sarcopenia, and osteosarcopenia

Sarcopenia was defined according to FNIH criteria as low MM associated with low HGS or low physical performance according to the GS and data for this sample already published elsewhere (22). Osteosarcopenia was deemed present in participants with sarcopenia plus low BMD (osteopenia or osteoporosis) or past self- reported low-energy trauma fractures after the age of 50 years (13).

### Biochemical evaluation

Laboratory data from participants in the T2DMG were collected from medical records (the last evaluation was considered when < 3 months) or from a fasting blood sample collected on the DXA evaluation day. Biochemical evaluation included fasting plasma glucose (normal range [NR] < 100 mg/dL), serum calcium (NR 8.5-10.2 mg/dL), glycated hemoglobin (HbA1c; National Glycohemoglobin Standardization Program, NR < 5.8% [International Federation of Clinical Chemistry 68 mmol/mol]), and serum parathyroid hormone (NR 15-68 pg/mL). Based on serum 25-hydroxyvitamin D levels measured by immunochemiluminescence (LIAISON, DiaSorin, Saluggia, Italy; CV ≤ 5%), the participants were classified as having normal (≥30 ng/mL), deficient (<20 ng/mL), or insufficient (21-29 ng/mL) vitamin D levels (23).

### Statistical analysis

All statistical analyses were performed using the R Core Team, v3.4.4 (R Foundation for Statistical Computing, Vienna, Austria). Normality was tested with the Kolmogorov-Smirnov test. Qualitative data are presented in absolute and relative frequencies and quantitative data as mean ± standard deviation or median (minimum and maximum) values. Quantitative variables were compared with Student’s *t* or Mann-Whitney test. Comparisons of three or more groups were made with the Kruskal-Wallis test and the *post hoc* test, while Fisher’s exact or chi-square test was used for qualitative analyses. Correlations between parametric variables were analyzed with Pearson’s correlation. Multivariate logistic regression was performed considering TBS as a dependent variable and as independent variables fractures, osteoporosis, osteopenia, BMI, high WC, low HGS, age, sex, weight, and BMD at all sites. Statistical significance was considered at p < 0.05.

## RESULTS

In the T2DMG, 442 patients with T2DM were screened and 265 were excluded, yielding 177 patients in this group as shown in Figure 1 (mean age 65.6 ± 8.6 years, 114 women). The CG comprised 146 individuals (mean age 65.0 ± 9.1 years, 80 women). More than 70% of the subjects in each group were older than 60 years. In all, 67.7% of the patients in the T2DMG were treated with insulin with or without oral agents. The mean dairy calcium intake was below the recommended amount in both groups and was lower in the T2DMG compared with the CG. Participants in the T2DMG compared with those in the CG also had more comorbidities and history of fractures (Table 1). CG had higher glomerular filtration rate and lower BMI than T2DMG.

**Table 1:** Clinical and laboratory characteristics of the study participants

			T2DMG (n = 177)	CG (n = 146)	P values
Age (years)			65.6 ± 8.6	65.0 ± 9.1	0.520
Sex					0.070
	Women		114 (64.4%)	80 (54.7%)	
	Men		63 (35.5%)	66 (45.2%)	
Ethnicity					
	Caucasians		149 (84.2%)	139 (95.2%)	0.440
	Mulattos/Blacks		26 (14.6%)	5 (3.42%)	<0.010
BMI (kg/m^2^)			29.2 ± 4.89	26.2 ± 3.10	<0.010
Waist circumference (cm)			99.7 ± 11.0	89.3 ± 10.0	<0.010
Menopause			111 (95.6%)	69 (86.2%)	0.090
Current smoking			12 (6.77%)	6 (4.10%)	0.720
Calcium intake (mg/day)			403 ± 3.5	651 ± 3.8	<0.010
Comorbidities					
	Dyslipidemia		154 (87.0%)	35 (27.7%)	<0.001
	Hypertension		145 (82.0%)	39 (30.3%)	<0.010
	Hypothyroidism		50 (28.2%)	16 (12.4%)	<0.001
Complications					
	Any complications		107 (60.4%)	N/A	
	Cardiovascular disease		53 (29.9%)	N/A	
	Retinopathy		52 (29.3%)	N/A	
	Distal motor sensory peripheral neuropathy		50 (28.2%)	N/A	
	Nephropathy		45.0 (25.4%)	N/A	
Biochemical analysis					
	Creatinine		1.18 ± 1.1	0.92 ± 0.23	<0.001
	Glomerular filtration rate (MDRD) mL/min/1.73 m^2^		71.0 ± 19.0	78.0 ± 15.0	<0.010
	Calcium mg/dL		9.41 ± 0.9	9.38 ± 0.47	<0.001
	PTH pg/mL		72.7 ± 50.5	N/A	N/A
	25-hydroxyvitamin D (ng/mL)		29.6 ± 13.3	N/A	N/A
		Deficient (<20 ng/mL)	80 (45.1%)		
		Insufficient (21 a 29 ng/mL)	47 (26.6%)		
		Sufficient (≥30 ng/mL)	50 (28.2%)		
	HbA1c		8.5% ± 5.7	N/A	N/A
Treatment of diabetes					
	Oral agent		56 (31.5%)	N/A	N/A
	Insulin only		22 (12.4%)	N/A	N/A
	Insulin plus oral agent		98 (55.3%)	N/A	N/A
History of fracture			53 (29.9%)	26 (18.5%)	0.001
ALM (kg)			20.2 ± 6.7	18.0 ± 4.1	0.001
ALM/IMC			0.70	0.69	0.810
TBS			1.272 ± 0.11	1.320 ± 0.12	0.001

Data are presented as mean ± standard deviation or frequency (percentage). T2DMG: type 2 diabetes mellitus group; CG: control group; BMI: body mass index; PTH: parathyroid hormone; HbA1c: glycated hemoglobin; TBS: trabecular bone score; TLM: total lean mass; ALM: appendicular lean mass; N/A: not available. P values < 0.05 were considered significant.

In the T2DMG, the mean duration of T2DM was 15.4 ±8.2 years, and the mean HbA1c level was 8.5 ± 5.7%. Overall, 66.6% of the participants in this group had an off-target HbA1c level (>7%) (24). Hypovitaminosis D and secondary hyperparathyroidism were observed in 80 (45.1%) and 46 (41.4%) patients, respectively, in the T2DMG. One-third of the T2DMG patients had macrovascular or microvascular complications (Table 1).

### Nonvertebral fractures

The T2DMG had more patients with a history of nonvertebral low-energy trauma fractures (n = 53, 29.9%) than the CG (n = 26, 18.5%; p = 0.001). The most common fracture sites were the legs, forearm, ankle, and wrist. When only patients with a history of fracture in each group were compared, the T2DMG had lower mean BMD values at all sites (p < 0.001 for all) and increased rates of osteoporosis (p = 0.003), distal motor sensory peripheral neuropathy (p = 0.043), and low HGS (p = 0.003).

### BMD, body composition, and TBS

Among men, total hip and lumbar spine BMD values and T-scores were greater in the T2DMG compared with the CG (p < 0.005 for all), while among women, BMD values at all sites were similar between groups (Figure 3). In addition, the rates of osteopenia and osteoporosis were comparable between groups, although values of BMI and TLM were greater in the T2DMG compared with the CG in both sexes (p < 0.001) (Table 1). Values of ALM were also greater in the T2DMG, but when ALM was corrected for BMI, the difference with the CG was no longer significant (Table 1).

**Figure 2:**
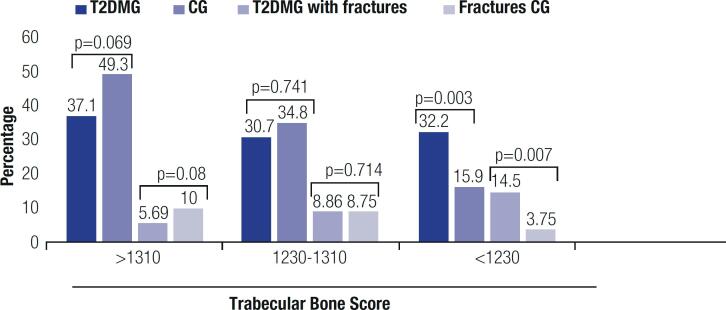
Trabecular bone score in T2DM patients and controls with and without a history of fractures. T2DMG: type 2 diabetes mellitus group; CG: control group.

TBS values were measured in 158 and 84 subjects in the T2DMG and CG, respectively. The mean TBS value was lower (1.272 ± 0.11) in the T2DMG compared with the CG (1.320 ± 0.12, p = 0.001). Additionally, the T2DMG had more patients with degraded TBS (p = 0.003) (Table 1, Figure 2).

**Figure 3:**
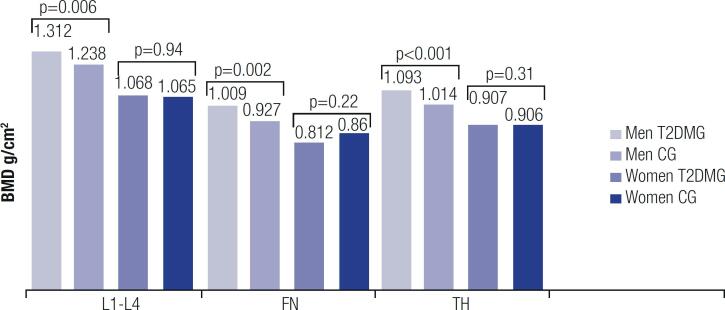
Bone mineral density in both groups. BMD: bone mineral density; T2DMG: type 2 diabetes mellitus group; CG: control group; FN: femoral neck; TH: total hip; L1-L4: lumbar spine 1 to 4.

In the T2DMG, the mean TBS was lower in Whites compared with Blacks (p = 0.016) and in patients with increased versus normal WC (p = 0.035), osteopenia (p = 0.003), or osteoporosis (p < 0.001) versus normal BMD, with versus without a fracture history (p = 0.009), and with versus without supplementation of calcium, vitamin D, or both (p < 0.001 for all). Of 46 patients in the T2DMG with a history of fracture who had TBS evaluated, 19 (41.3%) had degraded TBS (1.230 ± 0.108) and 14 (30.4%) had normal BMD.

In the T2DMG, TBS correlated negatively with age (r = -0.315, p < 0.001) and positively with weight (r = 0.408, p < 0.001), BMI (r = 0.195, p = 0.014), BMD T-scores at all sites (lumbar spine, r = 0.559, p < 0.001; femoral neck, r = 0.531, p < 0.001; and total hip, r = 0.564, p < 0.001), HGS (r = 0.400, p < 0.001), and GS (r = 0.273, p = 0.015). Mean TBS was associated to osteoporosis and fractures (p < 0.005) in both sexes. TBS had no correlation with other comorbidities or chronic diabetes complications.

On multivariate analysis using mean TBS as a dependent variable, and fractures, osteoporosis, osteopenia, BMI, increased WC, low TLM, low HGS, age, sex, weight, and BMD at all sites as independent variables, age, increased WC, history of fractures, lower TLM, and osteoporosis were associated with a high odds of having degraded TBS. In contrast, weight, male sex, and total hip BMD emerged as factors that confer a lower probability of having degraded TBS (Table 2).

**Table 2:** Variables associated with trabecular bone score on multivariate analysis

Predictors	Estimates	95% confidence interval	P value
Age	-0.004	-0.005 to -0.002	<0.001
Increased waist circumference	-0.065	-0.104 to -0.026	0.001
Fractures	-0.046	-0.078 to -0.015	0.004
Total lean mass	-0.008	-0.012 to -0.004	<0.001
Osteoporosis	-0.050	-0.095 to -0.004	0.032
Male	0.136	0.078 to 0.194	<0.001
Body mass index	0.005	0.003 to 0.007	<0.001
Total hip bone mineral density	0.116	0.019 to 0.212	0.019

### Evaluation of low TBS in T2DMG

In the T2DMG, 58 (36.7%) and 98 (62%) participants had low and normal TBS, respectively. Patients of both sexes with low TBS (partially and degraded, n = 98, 62.0%) compared with those with normal TBS (n = 58, 36.7%) were older (67.1 ± 9.0 years versus 63.1 ± 7.3 years, respectively, p < 0.005), more likely to have a history of fracture (n = 37, 38.9% versus n = 10, 16.1%; p = 0.004), a diagnosis of osteoporosis, and had lower BMD at all sites (p < 0.005). Men with low TBS had more frequently cataract (p = 0.029) and tended to have higher fasting glucose levels (p = 0.06) than those with normal TBS, while women with low TBS had higher PTH levels (p = 0.018) and took calcium and vitamin D supplements (p = 0.017) more frequently than those with normal TBS (Supplementary Table).

### Sarcopenia and osteosarcopenia

**Figure 4:**
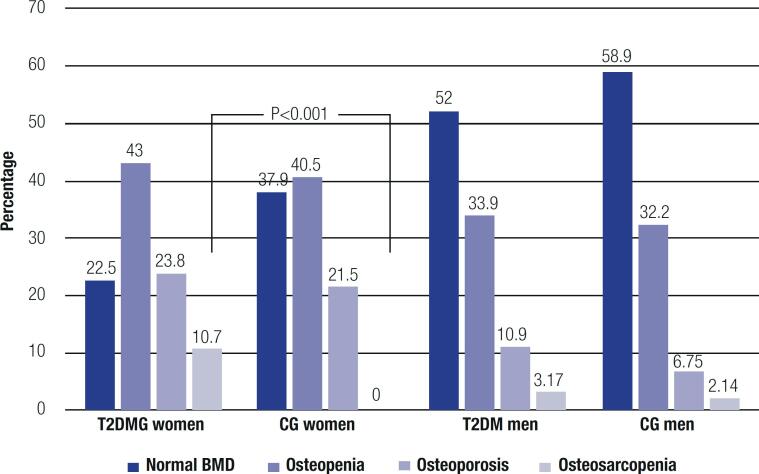
Prevalence of osteopenia, osteoporosis and osteosarcopenia. T2DMG: type 2 diabetes mellitus group; CG: control group; BMD: bone mineral density.

Sarcopenia was present in more subjects in the T2DMG (n = 23, 12.9%) than the CG (n = 8, 5.4%; p < 0.030) with no difference according to sex (p = 0.307) or ethnicity (p = 0.399), while osteosarcopenia was present in 21 (11.9%) participants in the T2DMG and 3 (2.14%) of those in the CG (p = 0.010). Osteopenia was a more frequent contributor to the diagnosis of osteosarcopenia than osteoporosis, as shown in Figure 4.

In women, osteosarcopenia was more prevalent in the T2DMG (n = 19 of 21) compared with the CG (n = 0 of 3; p = 0.002), while the distribution of men with osteosarcopenia was comparable between groups (p = 0.6870) (Figure 4). In the T2DMG, osteosarcopenia was associated with microvascular diabetes complications (p = 0.03), use of calcium and vitamin D supplements (p = 0.01), and leg fractures (p = 0.038). No association was observed between osteosarcopenia and HbA1c level (p = 0.719), fasting glucose level (p = 0.55), and disease duration (p = 0.09), or mean TBS value (p = 0.49).

## DISCUSSION

Our study found a higher prevalence of fractures, degraded TBS, sarcopenia, and osteosarcopenia in patients with T2DM compared with controls. The T2DMG comprised patients with long-term diabetes and HbA1c level above the recommended target, both of which have been previously associated with an increased risk of fractures (25).

The prevalence of osteosarcopenia was higher in participants with T2DM compared with controls. In a meta-analysis, rates of osteosarcopenia in elderly individuals varied between 5%-37% (26), but the prevalence of T2DM in the studies was unknown. The presence of osteosarcopenia in the T2DMG in the present study was associated with chronic diabetes complications but not with increased fasting glucose or HbA1c levels. The musculoskeletal unit involves mechanical, physical, and biochemical interactions via paracrine and endocrine communications. A link between the muscle-bone unit and diabetes is osteocalcin, a bone formation marker. Chronic hyperglycemia correlates inversely with circulating osteocalcin in patients with T2DM. Indeed, patients with T2DM have low circulating osteocalcin levels, which may affect bone structure and function (*e.g.*, increased fracture risk), reinforcing the link between skeletal and glucose metabolism (27).

The BMD measurement (the bone component of the osteosarcopenia) indicated a higher frequency of osteopenia compared with osteoporosis and a higher prevalence of osteopenia in women, which is aligned with the literature (28). In patients with T2DM, we found an association between fractures and peripheral neurosensitive motor neuropathy (often accompanied by decreased physical activity and increased falls), which may be a risk factor for osteoporosis, fractures, and sarcopenia (29). Men in the T2DMG had greater BMD than those in the CG, but the same was not found in women. This contrasts with data from the literature (30) and is probably explained by the older age and postmenopausal status of women in our study. Despite these findings, the T2DMG presented the so-called “diabetic paradox,” *i.e.*, more frequent fractures despite equal or greater BMD compared with the CG (31). This shows the importance of assessing TBS or other determinants of skeletal strength and fracture risk independent of BMD in patients with T2DM. Studies have shown that TBS is more degraded in patients with T2DM (32,33), which was confirmed in the present study. Our study also showed an increased rate of fractures in patients with degraded TBS (32-34).

Possibly due to the small sample size and the cross-sectional design of our study, we were unable to verify the association between lower TBS and poor glycemic control reported in other study (35). Degraded bone microarchitecture determined by TBS was present in one-third of our patients and was associated with fractures, osteopenia, and osteoporosis in both sexes, as shown in the literature, but not with osteosarcopenia (36).

Degraded TBS was associated with increased WC, confirming the harmful effect of visceral fat on bone, resulting in low BMD, worse bone microarchitecture, fractures and low TBS (37).

Variabilities in osteosarcopenia rates across different studies are mainly due to variations in the definition of sarcopenia. In the present study, we defined sarcopenia according to the FNIH criteria since these criteria correct the lean mass for body size, fitting better in our sample of overweight and obese patients. Recent initiatives by several international groups have attempted to homogenize the criteria to define sarcopenia, and muscle strength, most often measured by HGS, has become central to the definition. Muscle strength has been recognized as the best predictor of health outcomes in patients with or without diabetes (38). In our study, low HGS was associated with a greater risk of nonvertebral fractures, degraded TBS, and osteosarcopenia, which is aligned with the literature (39). This association may be due to fat infiltration in muscle and bone tissue with consequent osteoporosis and reduced strength; fat infiltration may occur with age or result from chronic inflammation in obesity and T2DM (40).

Limitations of our study include the small sample size, reduced number of men and CG were matched only by age and sex with T2DMG. Also, the study’s cross-sectional design does not allow the evaluation of a cause-effect relationship. Strong points of the study are the inclusion of a control group, use of DXA to assess BC, measurement of HGS in all patients and controls, and analysis of fractures and osteosarcopenia in both T2DMG and CG.

In conclusion, patients with T2DM had a higher prevalence of osteosarcopenia and degraded TBS compared with controls. Osteosarcopenia was associated with diabetes complications but not with diabetes duration or glycemic control.

Population aging is increasing the need for care. Prevention, early detection, and better treatment of sarcopenia and osteoporosis in patients with T2DM will be necessary to meet this population’s demands in the future.

## Appendix

**Supplementary Table:** Variables associated with low or normal trabecular bone score in patients with type 2 diabetes mellitus

Variables		Low TBS	Normal TBS	P value
***Women***				
BMI (kg/m^2^)		8.5 ± 4.7	30.8 ± 5.5	0.064
WC (cm)		96.7 ± 12.4	100.5 ± 11.01	0.154
HbA1c		7.95 ± 1.8	8.08 ± 1.5	0.526
Glycemia (mg/dL)		133 ± 61	147 ± 91	0.25
PTH (pg/mL)		78.5 ± 52.7	50.2 ± 24.7	0.018
Calcium (mg/dL)		9.4 ± 1.1	9.3 ± 0.8	0.547
Calcium and vitamin D supplementation		42 (57.5%)	8 (28.5%)	0.017
History of fracture		26 (35.6%)	3 (10.7%)	0.014
BMD (g/cm^2^)				
	Lumbar spine	1.037 ± 0.17	1.172 ± 0.16	0.001
	Femoral neck	0.762 ± 0.5	0.938 ± 0.13	<0.001
	Total hip	0.901 ± 0.16	1.031 ± 0.12	<0.001
Osteopenia		37 (50.6%)	12 (46.1%)	0.866
Osteoporosis		20 (27.3%)	1(3.84%)	0.011
ALM (kg)		17.1 ± 4.7	17.6 ± 2.9	0.366
Low muscle mass		7 (26.9%)	16 (22.5%)	0.788
HGS (kg)		19.4 ± 6.0	19.6 ± 5.9	0.858
GS (m/s)		1.030 ± 0.29	1.132 ± 0.32	0.288
***Men***				
BMI (kg/m^2^)		29.4 ± 4.04	28.08 ± 4.48	0.220
WC (cm)		100.8 ± 11.0	103.4 ± 9.1	0.383
HbA1c		8.45 ± 1.6	8.04 ± 1.7	0.373
Glycemia (mg/dL)		181 ± 73	150 ± 54	0.06
Calcium (mg/dL)		9.3 ± 0.45	9.3 ± 0.67	0.981
Cataract		5 (22.7%)	1 (2.94%)	0.029
History of fracture		11 (50%)	7(20.5%)	0.044
BMD (g/cm^2^)				
	Lumbar spine	1.227 ± 1.20	1.368 ± 1.3	0.013
	Femoral neck	0.917 ± 0.15	1.046 ± 0.12	0.002
	Total hip	0.982 ± 0.25	1.149 ± 0.12	0.001
	Osteopenia	12 (57.1%)	8(52.9%)	0.025
Osteoporosis		3 (14.2%)	1 (2.94%)	0.150
ALM (kg)		23.3 ± 2.5	26.2 ± 8.9	0.305
Low muscle mass		7 (33.3%)	10 (29.4%)	0.772
HGS (kg)		32.2 ± 13.77	34.45 ± 6.88	0.081
GS (m/s)		1.32 ± 0.29	1.28 ± 0.34	0.736

Data are presented as mean ± standard deviation or frequency (percentage). BMI: body mass index; WC: waist circumference; PTH: parathyroid hormone; HGS: handgrip strength; GS: gait speed; BMD: bone mineral density; ALM: appendicular lean mass. Low muscle mass was defined as ALM/BMI < 0.789 in men and < 0.512 in women.
